# Dry Mixing Process Optimization of High-Viscosity Permeable Asphalt Mixtures Using the Response Surface Methodology (RSM) and Investigation of the Mixing Mechanism

**DOI:** 10.3390/ma19101946

**Published:** 2026-05-09

**Authors:** Zehua Chen, Longfei Mei, Dali Zhang, Jiajun Ji, Xiaoyi Ban, Zengping Zhang

**Affiliations:** 1 Key Laboratory for Special Area Highway Engineering of Ministry of Education, Chang’an University, Xi’an 710064, China; zczqzzc@ucl.ac.uk (Z.C.); mlf101110@163.com (L.M.); 2025021079@chd.edu.cn (J.J.); 2021021087@chd.edu.cn (X.B.); 2 Bartlett School of Planning, University College London, London WC1E 6BT, UK; 3 Shandong Expressway Infrastructure Construction Co., Ltd., Jinan 250099, China; zsirli@163.com

**Keywords:** response surface methodology (RSM), permeable asphalt mixture, dry process, HVM (high-viscosity modifier), mixing parameter optimization, mixing mechanism

## Abstract

To optimize the dry mixing preparation process for permeable asphalt mixtures and elucidate the microscopic mechanism of high-viscosity modifiers, this study employed Response Surface Methodology (RSM). Independent variables, including mixing temperature, dry mixing time, and wet mixing time, were selected, with Marshall stability, −10 °C splitting tensile strength, and residual stability as response indicators. The significance and fitting accuracy of the model were evaluated through Analysis of Variance and residual diagnosis. Additionally, the morphological evolution, spatial distribution, and chemical interactions of the modifier were characterized using scanning electron microscopy (SEM), fluorescence microscopy (FM), and Fourier Transform Infrared Spectroscopy (FTIR). The results revealed distinct differences in factor effects: mixing temperature most strongly influenced high-temperature performance, while wet-mixing time primarily governed low-temperature performance and water stability. Furthermore, significant interactions were observed among the variables. Multi-objective optimization determined the optimal process parameters to be a mixing temperature of 182 °C, dry mixing time of 180 s, and wet mixing time of 102 s. Experimental validation confirmed that the relative error between predicted and actual values was within 5%. Microscopic characterization revealed that under the dry mixing process, the modifier undergoes four stages: rapid melting, viscous flow, permeation diffusion, and swelling development. An appropriate mixing temperature combined with sufficient dry and wet mixing times promotes the formation of a uniform, dense spatial network structure of the modifier within the asphalt. This study validated the reliability of RSM in process optimization, providing theoretical foundations and key technical parameters for the green and efficient production of high-performance permeable asphalt mixtures.

## 1. Introduction

With accelerating global climate change and rapid urbanization, the demand for road safety, environmental sustainability, and driving comfort has intensified, leading to the widespread adoption of permeable asphalt pavements. These pavements are characterized by a connected air void content of 18% to 25%, which facilitates rapid drainage of surface runoff and enhances driving safety under wet conditions [1,2]. In addition to drainage capacity, permeable asphalt mixtures provide multifunctional benefits, including noise reduction and thermal regulation, which help mitigate traffic noise and the urban heat island effect while maintaining excellent skid resistance [3,4,5]. However, the high air void content and significant proportion of coarse aggregates render the mixture susceptible to oxidative aging and stripping under the coupled effects of environmental factors, leading to various pavement distresses [6,7]. Therefore, employing high-viscosity asphalt as the binder is crucial for ensuring the long-term durability and service performance of porous asphalt pavements [8,9,10]. Despite these advantages, the performance of high-viscosity asphalt mixtures is strongly dependent on the preparation process, particularly the mixing method and process parameters, which directly control the dispersion and effectiveness of the modifier.

Conventionally, high-viscosity asphalt is prepared via the wet process, involving the pre-blending of modifiers with base asphalt under high-temperature and high-shear conditions prior to mixture production [11,12]. Although this method ensures uniform modifier dispersion, it has several limitations, including poor storage stability, susceptibility to phase separation during transport, high energy consumption, and reliance on specialized equipment [13,14,15]. Compared to the wet method, the dry process involves initially dry mixing the granular HVM with aggregates to ensure uniform distribution. This mixture is then wet-mixed with base asphalt and mineral filler to produce the drainage asphalt mixture. The shearing action of the aggregates facilitates the in situ dispersion and development of the modifier. This mechanism eliminates the need for prolonged mixing and shear modification, bypassing the production, transportation, and storage steps associated with high-viscosity asphalt, thereby significantly streamlining the process [16]. Through this highly intensive mixing, multiple physicochemical modification reactions are achieved simultaneously within a matter of seconds. Although the dry process demonstrates significant application potential, its core mixing parameters have a decisive impact on the final mixture’s performance [17,18].

Existing research indicates that insufficient mixing temperatures or excessively short mixing times may prevent the complete melting and dispersion of modifier particles, significantly reducing modification effectiveness. Conversely, excessively high temperatures or prolonged mixing times can cause asphalt oxidative aging or modifier degradation, similarly compromising performance [19,20]. These observations suggest the existence of an optimal parameter window, making systematic optimization essential. However, current research faces two major challenges. First, there are notable limitations in existing experimental design methods. Traditional studies predominantly employ one-factor-at-a-time methods or orthogonal experimental designs. For instance, Yu et al. [21] investigated high-modulus asphalt concrete by sequentially determining the dry mixing time, mixing temperature, and wet mixing time of the modifier to establish preparation parameters. Xiao et al. [22] employed an L9(3^3^) orthogonal array to conduct range analysis on mineral filler addition timing, mixing temperature, and total mixing time for gussasphalt mixtures. While successfully ranking the significance of factors, this approach failed to predict optimal process points between the discrete levels. Similarly, Zhao et al. [23] optimized the preparation process for rubber-modified asphalt mixtures using orthogonal design. Range analysis identified “digestion time” as the primary influencing factor and determined an optimal combination. However, this method cannot construct a continuous response surface model that accounts for the interactions among process parameters. While these methods can preliminarily identify primary influencing factors, they fail to precisely quantify the interactions between them. Consequently, process optimization often remains confined to local optima, failing to achieve the best balance of overall mixture performance. Second, existing studies primarily focus on macroscopic performance, with limited attention to the microstructural evolution of modifiers and their interfacial interactions with the asphalt binder during the dry mixing process. This gap restricts the development of a mechanistic understanding and limits theoretical guidance for process optimization [24,25]. Response Surface Methodology (RSM) is a crucial mathematical and statistical approach for process optimization and experimental design. Its significant advantages include reducing the number of experimental runs, shortening research cycles, and achieving high precision [26,27]. Consequently, it has been widely applied to the mix design of modified asphalt and asphalt mixtures, as well as the optimization of process parameters [28,29,30,31]. However, its application in dry-mix high-viscosity asphalt systems remains limited, particularly in terms of integrating statistical optimization with microstructural mechanism analysis.

From the perspective of interfacial mechanics, mixing temperature and dry mixing time directly influence the formation of the modifier film on aggregate surfaces. Subsequently, during the wet mixing stage with the base asphalt, physical or chemical changes occur at the interface between the asphalt and modifier films, locally reinforcing the binder matrix [32,33,34]. Temperature provides the critical thermal energy to overcome the modifier’s activation barriers for melting and flow, while sufficient time is essential for the kinetic processes of film spreading and polymer network swelling to reach completion. An imbalance between these two parameters directly compromises the modification efficiency and final performance.

In this study, a Response Surface Methodology (RSM) framework is employed to investigate the dry mixing process of permeable asphalt mixtures incorporating a laboratory-prepared high-viscosity modifier (HVM). Three key process variables—mixing temperature, dry mixing time, and wet mixing time—are selected based on their direct influence on modifier activation and dispersion. Other factors, such as binder content and aggregate gradation, are controlled to isolate the effects of process parameters. The effects of these variables on Marshall stability, splitting tensile strength at −10 °C, and residual stability are systematically analyzed. In addition, Scanning Electron Microscopy (SEM) and Fluorescence Microscopy (FM) are used to characterize the morphological evolution of the modifier, while FTIR analysis is conducted to examine its interaction with the asphalt binder. Through this integrated approach, the study not only identifies optimal process parameters but also provides an evidence-based interpretation of modifier evolution during the dry mixing process. Therefore, this study provides not only a set of optimized technical parameters but, more importantly, a robust scientific foundation and a validated methodological framework for the green and efficient production of high-performance permeable asphalt mixtures using the dry-mix technology.

## 2. Materials and Methods

### 2.1. Materials

#### 2.1.1. High-Viscosity Modifier

**Figure 1:**
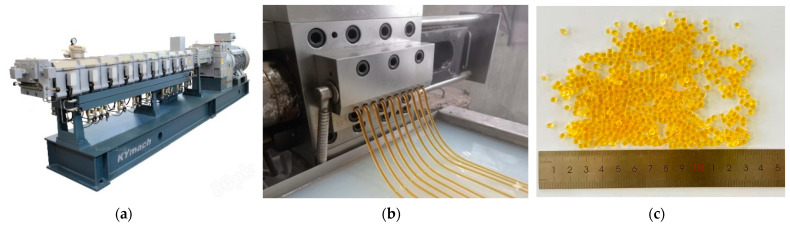
Preparation of HVM: (**a**) Twin-screw extruder; (**b**) HVM production process; (**c**) High-viscosity modifiers.

Due to the characteristics of SBS, the dosage level can affect the performance of polymer-modified bitumen [35], the high-viscosity modifier was prepared in the laboratory using linear SBS with an S/B block ratio of 40:60 (6 wt% of the bitumen) and a viscosity-enhancing resin (TP) at 4 wt% of the bitumen as the main raw materials, with an organic ester compound of 99.5% purity added as a plasticizer. The production process and the resulting granules are illustrated in Figure 1. The technical specifications of the modifier are listed in Table 1. The HVM is added separately, at a dosage of 12% by weight of the bitumen.

#### 2.1.2. Performance Evaluation of High-Viscosity Modifiers

In order to better analyze the mechanism of action and the role of modifiers in dry-process permeable asphalt mixtures, this study investigated the melting rate, molecular structure and thermal stability of high-viscosity modifiers, based on the selection of raw materials.

(1) Melt Mass Flow Rate (MFR)

This study strictly adhered to Plastics-Determination of the melt mass-flow rate (MFR) and melt volume-flow rate (MVR) of thermoplastics-Part 1: Standard method (GB/T 3682.1-2018) [37]. The melt flow rate tester used was the QT-400A model manufactured by Suzhou Qiantong Instrument Equipment Co., Ltd. (Suzhou, China). The test temperature was set at 190 °C, with a nominal load of 2.16 kg. The MFR value of the high-viscosity modifier was calculated using Equation (1), and the test results are detailed in Table 2.

MFR (T,m_nom_) = 600 × m/t (1)

In the formula: T represents the test temperature (°C); m_nom_ represents the nominal load (kg); m represents the average mass of the cut section (g); 600 represents the conversion factor from g/s to g/10 min; t represents the time interval between cuts (s).

As can be seen from Table 2, the melt mass flow rate of the high-viscosity modifier meets the technical requirements specified in the standard.

(2) Differential Scanning Calorimetry (DSC), Thermogravimetry (TG) and Fourier-transform Infrared Spectroscopy (FTIR) Analysis

**Figure 2:**
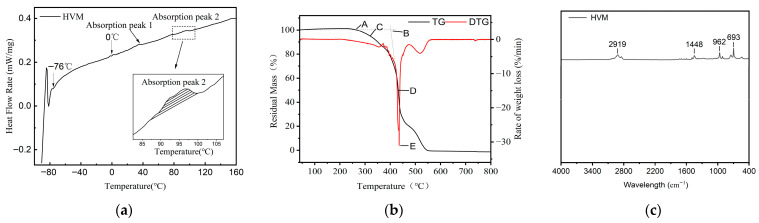
DSC, TG and FTIR analysis: (**a**) DSC curves of high-viscosity modifiers; (**b**) TG-DTG curves of high-viscosity modifiers; (**c**) Infrared spectrum of high-viscosity modifiers.

This experiment utilized a 200F3 differential scanning calorimeter manufactured by NETZSCH-Gerätebau (Shanghai, China). The thermal flux variation curve of the modifier, with material temperature on the x-axis and heat flux (dW/dt) on the y-axis, is shown in Figure 2a. A TG-209F3 thermal gravimetric analyzer, also manufactured by the same company, was used to perform thermal-oxygen analysis on the high-viscosity modifier. The thermogravimetric curve of the modifier is shown in Figure 2b. A Vertex 70 Fourier transform infrared spectrometer manufactured by Bruker, a German company, in Beijing, China was used. Infrared scans were conducted on the high-viscosity modifier, the test results and the functional groups corresponding to each absorption peak are shown in Figure 2c and Table 3, respectively.

Figure 2a shows that, with increasing temperature, the DSC curves of the high-viscosity modifiers rise rapidly and then gradually level off, exhibiting a generally similar trend. The modifiers display one or more glass transition temperatures within the low-temperature range of −80 °C to −40 °C, indicating the presence of incompatible components. At room temperature, these components remain highly elastic, contributing to fatigue resistance and low-temperature flexibility. In addition, phase transitions within the polymer system are reflected as endothermic peaks in the DSC curves. With increasing temperature, the melting of polymer crystals produces distinct endothermic peaks. In the medium-to-high temperature range, multiple endothermic peaks are observed, which are likely associated with the melting of different components within the modifier. In particular, a pronounced endothermic peak appears between 80 °C and 115 °C, corresponding to the melting of thermoplastic elastomer phases. At ambient conditions, these phases act as physical cross-linking points that enhance mechanical strength and deformation resistance. Once the melting temperature is reached, the network structure weakens, and the polymer chains soften and begin to flow, which facilitates subsequent mixing and modification of the bitumen.

In Figure 2b, point A represents the onset temperature (the temperature at which the TG curve begins to deviate from the initial baseline); point B represents the onset temperature of exothermic decomposition (the temperature corresponding to the intersection of the initial baseline and the tangent to the descending portion of the TG curve); point C represents the temperature at which the material has lost 5% of its mass; point D represents the temperature at which the material has lost 50% of its mass; and point E represents the temperature at which the maximum rate of weight loss on the DTG curve is observed. Figure 2b indicates that the initial decomposition temperature of the HVM is approximately 270 °C, which is higher than typical asphalt mixing temperatures. Although some oxidation and decomposition of additives (e.g., plasticizers) may occur under elevated temperatures, significant degradation of the polymer components does not occur until temperatures exceed 350 °C. Therefore, the thermal stability of the high-viscosity modifier is sufficient for asphalt mixture production.

As shown in Figure 2c and Table 3, all three modifiers exhibit characteristic absorption peaks at 2919 cm^−1^, corresponding to the asymmetric stretching vibration of methylene groups (–CH_2_), and at 1448 cm^−1^, associated with aromatic C=C skeletal vibrations. In the fingerprint region, the peaks at 962 cm^−1^ and 693 cm^−1^ correspond to out-of-plane bending vibrations of trans- and cis-alkene groups, respectively. The presence of absorption peaks in the range of 730–693 cm^−1^ indicates that the modifiers contain long-chain alkene structures.

(3) Melting characteristics of high-viscosity modifiers in dry-mix applications

**Figure 3:**
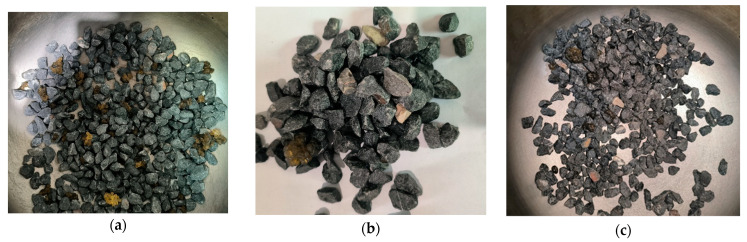

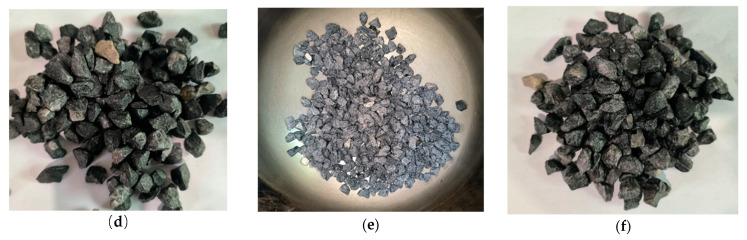
Melting behavior of HVM on aggregate surfaces at different mixing temperatures and dry mixing times: (**a**) Dry mix at 160 °C for 90 s; (**b**) Dry mix at 160 °C for 180 s; (**c**) Dry mix at 170 °C for 90 s; (**d**) Dry mix at 170 °C for 180 s; (**e**) Dry mix at 180 °C for 90 s; (**f**) Dry mix at 180 °C for 180 s.

Figure 3 shows that mixing temperature and dry mixing time significantly affect the melting behavior and distribution uniformity of the modifier. At 160 °C, even with a dry mixing time of 180 s, most of the modifier remains unmelted, with only partial softening and agglomeration observed. At 170 °C, melting becomes more pronounced; however, viscous flow is only achieved after extended mixing (180 s), resulting in discontinuous and uneven films. At 180 °C, the modifier is largely melted after 90 s, although relatively thick films remain. When the mixing time is extended to 180 s, the modifier flows more uniformly and forms a thin, continuous coating on the aggregate surface. This increases the effective contact area and promotes better interaction with the base bitumen during subsequent wet mixing, thereby improving modification effectiveness.

#### 2.1.3. Aggregate and Asphalt

Basalt was used as the coarse aggregate, while manufactured basalt sand served as the fine aggregate, and limestone mineral powder was used as the filler. All tests were conducted in accordance with the Test Methods of Highway Engineering (JTG 3432–2024) [38]. The main performance index test results are presented in Table 4, Table 5 and Table 6. Test results confirmed that all aggregates and the filler met the relevant technical requirements. Since high-viscosity modifiers are primarily composed of polymers such as thermoplastic elastomers and resins, their modification effects vary depending on the type of base asphalt [39]. Therefore, this study selected Grade A 70# petroleum asphalt from Shandong Chambroad Petrochemical Co., Ltd. (Binzhou, China). as the base binder. Its fundamental properties were tested in accordance with the requirements of the Testing Procedures for Asphalt and Asphalt Mixtures in Highway Engineering (JTG 3410-2025) [40]. The test results and corresponding specification requirements are presented in Table 7.

### 2.2. Methods

#### 2.2.1. Design of Mixture Proportions for Permeable Asphalt Mixture

**Figure 4:**
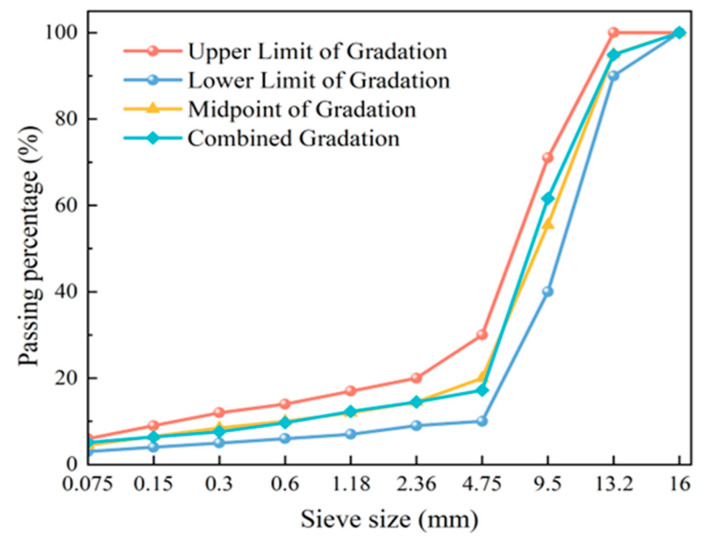
Gradation curve of permeable asphalt mixture.

Based on engineering practice, this study adopted a PA-13 gradation design, with the gradation curve illustrated in Figure 4. Following the test methods T 0732 and T 0733 specified in the Testing Procedures for Asphalt and Asphalt Mixtures in Highway Engineering (JTG 3410-2025) [40], the optimum binder-to-aggregate ratio for the high-viscosity asphalt mixture was determined to be 5.0%. At this ratio, the binder drain-down was ≤0.8% and the Cantabro loss was ≤8.6%, both satisfying the standard requirements.

#### 2.2.2. Design of Experiments: Factors and Levels

Selection of factors and their corresponding levels was based on mechanistic considerations and practical constraints in asphalt mixture production. Mixing temperature, dry mixing time, and wet mixing time were identified as the most critical variables in the dry mixing process. The performance of dry-mix asphalt mixtures depends on the in situ melting, dispersion, and swelling of the HVM, which are primarily influenced by thermal conditions and mixing duration.

The levels for each factor were selected to be both scientifically meaningful and practically relevant, based on preliminary experimental observations. These results showed that the modifier exhibited insufficient melting below 170 °C, even when the dry mixing time was extended to 270 s, thereby establishing a practical lower bound. As temperature and mixing time increased, the melting behavior of the modifier and the homogeneity of the mixture improved progressively. Therefore, the mixing temperature range was set at 170–190 °C to balance effective melting with the risk of oxidative aging. The dry mixing time was set at 90–270 s to capture this transition. These ranges, together with a wet mixing time of 60–120 s, are based on established industry practices. They are sufficiently wide to capture key performance variations while remaining feasible for plant implementation without significantly reducing productivity.

#### 2.2.3. Determination of Response Values

Due to their high void content, permeable asphalt pavements are more susceptible to environmental factors such as moisture and temperature fluctuations, resulting in reduced high-temperature stability, increased cracking potential, and lower moisture resistance. Therefore, in the response surface methodology (RSM) analysis, Marshall stability (R_1_), splitting tensile strength at −10 °C (R_2_), and residual stability (R_3_) were selected as response variables to optimize the dry-mix process.

It should be noted that other important performance indicators for permeable asphalt mixtures, such as Cantabro loss and binder drain-down, were not included as response variables in the RSM optimization. This is because these parameters were already used during the mixture design stage to determine the optimal binder content while ensuring compliance with specification requirements. Therefore, in this study, Cantabro loss and drain-down were treated as design constraints rather than optimization objectives. By fixing the binder content based on these criteria, the RSM analysis focuses on the influence of process parameters on mechanical performance. Future studies may incorporate these indicators into a multi-objective optimization framework to provide a more comprehensive evaluation.

#### 2.2.4. Response Surface Design

A Face-Centered Central Composite Design (CCF) [41] was employed to optimize the dry mixing process. Mixing temperature (A), dry mixing time (B), and wet mixing time (C) were selected as the independent variables, each evaluated at three levels. The specific experimental design is presented in Table 8. Marshall stability (R_1_), splitting tensile strength at −10 °C (R_2_), and residual stability (R_3_) served as the response variables for optimizing the permeable asphalt mixtures.

#### 2.2.5. Dry Mixing Process for Permeable Asphalt Mixture

**Figure 5:**
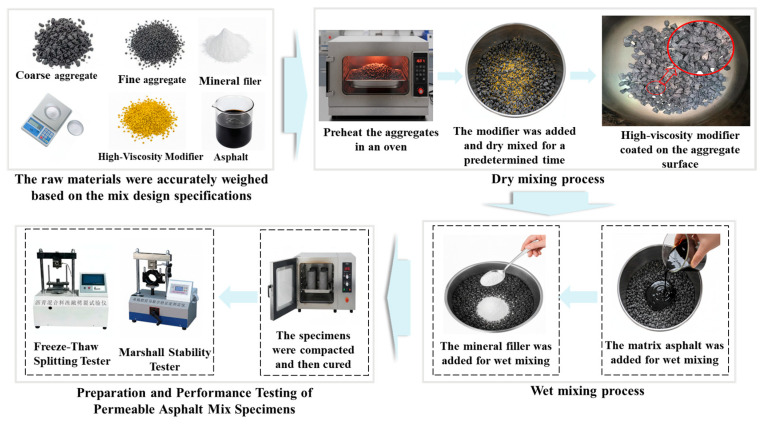
Flowchart of dry mixing process for permeable asphalt mixture (The red circle indicates the high-viscosity modifier coating the surface of the aggregate).

The dry mixing process for the permeable asphalt mixture is illustrated in Figure 5. This process primarily consists of three key sequential stages. In the first stage, heated aggregates are introduced into the mixing drum and combined with a specified amount of high-viscosity modifier (HVM). In the second stage, base asphalt is added to the mixture obtained from the dry mixing stage to initiate wet mixing. In the final stage, mineral powder is incorporated to complete the mixture preparation.

#### 2.2.6. Pavement Performance Evaluation

All tests were conducted in accordance with the Testing Procedures for Asphalt and Asphalt Mixtures in Highway Engineering (JTG 3410-2025). Specimens were prepared accordingly, and their high-temperature performance, low-temperature behavior, and water stability were evaluated. Each test was conducted at least three times [40].

(1) High-temperature stability test

To evaluate the high-temperature performance of asphalt mixtures, Marshall stability tests were conducted in accordance with T0709-2025. Prior to testing, three specimens were immersed in a 60 °C water bath for 30 to 40 min. The Marshall stability (MS) values were recorded for each specimen. A higher MS value indicates superior resistance to permanent deformation at elevated temperatures.

Marshall stability is used in this study due to its simplicity and widespread application in laboratory evaluation. However, it primarily reflects load-bearing capacity under quasi-static conditions. It may not fully capture rutting resistance under repeated loading. Therefore, the results should be interpreted with caution when extrapolated to field performance.

Despite this limitation, the selected indicators allow for comparative analysis and provide a practical basis for process optimization within the scope of this study.

(2) Low-temperature crack resistance test

To assess the low-temperature crack resistance of asphalt mixtures, a splitting test was conducted at −10 °C in accordance with T0716-2025, with a constant loading rate of 1 mm/min. Prior to testing, specimens were conditioned at −10 °C for at least 6 h to ensure thermal equilibrium. The splitting tensile strength was measured, with higher values indicating superior resistance to low-temperature cracking.

(3) Water stability test

The water stability of asphalt mixtures was evaluated by determining the residual stability, with the specific method referenced in T0709-2025. Three specimens were immersed in a 60 °C constant-temperature water bath for 30 min and three specimens for 48 h, respectively. The Marshall stability after 30 min of immersion and after 48 h of immersion were measured. Residual stability was calculated as:

MS_0_ = MS_1_/MS × 100 (2)

where MS_0_ is the residual stability (%), MS_1_ is the Marshall stability after 48 h immersion, and MS is the Marshall stability after 30 min immersion (kN).

#### 2.2.7. Microstructural Characterization Methods

(1) Scanning electron microscope (SEM) test

The microstructure of the dry-mixed aggregates and the resulting mixtures was characterized using an analytical thermal field emission scanning electron microscope (MLA FEG 650). To facilitate sample preparation, particles passing the 4.75 mm sieve were randomly selected for analysis. For the SEM observations, an accelerating voltage of 10–15 kV was applied. The working distance was controlled within 8–10 mm, and both secondary electron (SE) and backscattered electron (BSE) modes were employed to capture surface morphology and phase contrast. Images were obtained at multiple magnifications, including 1000×, and 3000×, to ensure both global morphology and local structural features were adequately represented. Given the non-conductive nature of the modifier, aggregates, and mixture, the samples were sputter-coated with gold prior to imaging to prevent surface charging and ensure high image quality.

(2) Fluorescence microscopy (FM) test

**Figure 6:**
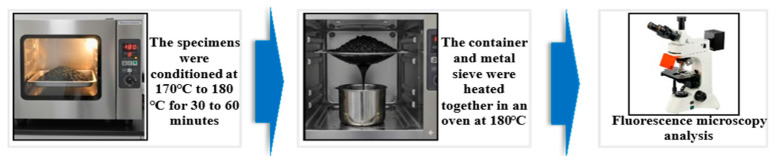
High-viscosity asphalt extraction process.

Using the LW300LFT epifluorescence microscope from Shanghai Cewei Optoelectronic Technology Co., Ltd. (Shanghai, China), the distribution state of high-viscosity modifier in asphalt under different mixing conditions was investigated. For fluorescence microscopy (FM), observations were conducted under consistent excitation conditions, and images were captured at magnifications of 400×. As illustrated in Figure 6, the high-viscosity asphalt was extracted from the mixture through a specific three-step procedure. Initially, the dry-process permeable asphalt mixture was conditioned at 170 °C to 180 °C for a duration of 30 to 60 min. Subsequently, the mixture was placed in a metal sieve with a container positioned underneath to collect the dripping binder, and the entire assembly was transferred to an oven maintained at 180 °C. Finally, as the asphalt underwent viscous flow due to heating, an appropriate quantity of the high-viscosity asphalt dripping into the metal container was collected for analysis.

(3) Fourier Transform Infrared Spectroscopy (FTIR) test

A Vertex 70 Fourier transform infrared (FTIR) spectrometer (Bruker, a German company, in Beijing China) was used to analyze the molecular composition of the bituminous binder. The bitumen samples were prepared using the pellet method. Each test was conducted with 32 scans over a wavenumber range of 4000–400 cm^−1^. Spectra were collected for the base bitumen, the high-viscosity modifier (HVM), and the binders extracted under different mixing conditions.

(4) Differential Scanning Calorimetry (DSC), Thermogravimetry (TG) test

A DSC 200F3 differential scanning calorimeter and a TG 209F3 thermogravimetric analyzer (both manufactured by NETZSCH-Gerätebau, Shanghai, China) were used to evaluate the thermal properties of the high-viscosity modifier.

For DSC analysis, the heating rate was 10 °C/min under a nitrogen atmosphere with a flow rate of 20 mL/min, and the temperature range was −90 °C to 160 °C.

For TG analysis, the temperature range was 40 °C to 800 °C, with a heating rate of 10 °C/min under an air atmosphere at a flow rate of 50 mL/min.

**Figure 7:**
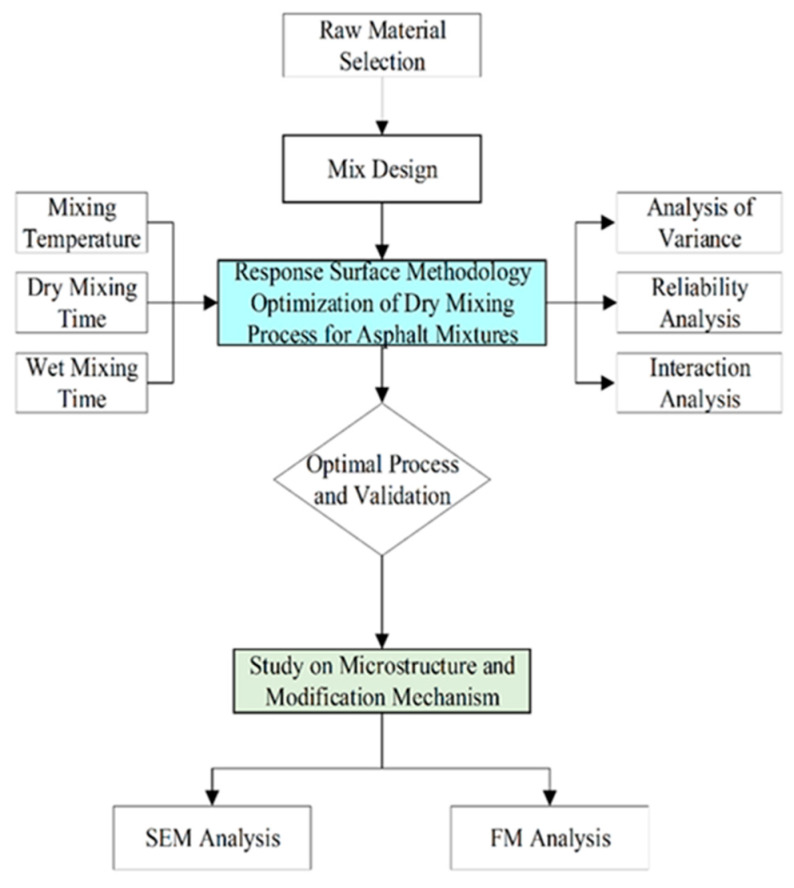
Flowchart of the experimental design and analysis process for dry-mix asphalt mixtures.

To provide a comprehensive overview of the experimental procedures and analytical workflow, the technical roadmap for this study is presented in Figure 7.

## 3. Results

### 3.1. Experimental Results

Design-Expert software was utilized to conduct a three-factor, three-level analysis on the aforementioned parameters [42]. Based on this, The CCF optimization scheme for the dry mixing process was determined. The experimental design comprised 17 runs, and each response value was obtained as the average of three specimens. The CCF design scheme and the corresponding results are detailed in Table 9. The results show that R_1_, R_2_, and R_3_ vary significantly across different factor combinations, indicating strong nonlinear behavior. This suggests complex interactions among the control factors in the dry process, providing a robust data foundation for the subsequent fitting of quadratic regression models and multi-objective parameter optimization. Additionally, the experiment included multiple sets of center-point replicates (e.g., runs 1, 5, and 8). The variance of the responses at these points was used to evaluate the stability and reproducibility of the experimental procedure.

### 3.2. Model Variance Analysis

Following the experiments, the data were subjected to regression analysis. Analysis of variance (ANOVA) was performed to evaluate the goodness-of-fit for the regression model of each response. The ANOVA results are summarized in Table 10. As shown, the *p*-values for all three models are less than 0.05, indicating that they are statistically significant. Furthermore, the high values of the adjusted determination coefficient (R^2^adj) confirm that the models provide an excellent fit to the experimental data.

For R_2_ and R_3_, the high values of adjusted R^2^ and predicted R^2^, along with their close agreement, suggest that the models possess good explanatory power and predictive reliability. This indicates that the selected variables effectively capture the variation in low-temperature performance and water stability. However, for R_1_, a noticeable discrepancy between the adjusted R^2^ and predicted R^2^ is observed. The relatively low predicted R^2^ suggests limited predictive capability of the model for this response. This may be attributed to the inherent variability of the Marshall test or the insufficient sensitivity of this indicator to capture subtle changes in process parameters.

Therefore, while the model for R_1_ is statistically significant, its predictive robustness should be interpreted with caution. Future research should incorporate additional performance metrics to enhance the model’s reliability.

Based on the analysis of variance (Table 10), the final predictive models for Marshall stability (R_1_), low-temperature splitting strength (R_2_), and residual stability (R_3_) were established. The regression equations are presented in Equations (3)–(5), respectively.

R_1_ = −142.94333 + 1.67329x_1_ + 0.01303x_2_ − 0.094406x_3_ − 0.00002x_1_x_2_ + 0.000639x_1_x_3_ 
− 2.16049 × 10^−6^x_2_x_3_ − 0.004755x_1_^2^ − 0.000026x_2_^2^ − 0.000123x_3_^2^ (3)

R_2_ = −34.87937 + 0.408669x_1_ + 0.002856x_2_ + 0.007376x_3_ + 2.77778 × 10^−6^x_1_x_2_ + 5.77827 × 10^−18^x_1_x_3_ + 9.25926 × 10^−7^x_2_x_3_ − 0.001134x_1_^2^ − 0.905929 × 10^−6^x_2_^2^ − 0.000037x_3_^2^ (4)

R_3_ = −222.56747 + 3.28796x_1_+ 0.091207x_2_ + 0.164108x_3_ + 0.00018x_1_x_2_ − 0.00038x_1_x_3_ − 0.00014x_2_x_3_ − 0.009002x_1_^2^ − 0.000301x_2_^2^ − 0.000269x_3_^2^ (5)

where x_1_ is the mixing temperature (°C), x_2_ is the dry mixing time (s), and x_3_ is the wet mixing time (s).

Overall, the regression models provide a useful tool for understanding factor interactions and identifying optimal parameter ranges, but their predictive application should be validated with additional experimental data.

A reliability analysis was conducted on the quadratic polynomial regression models for the three response values; the results are shown in Table 11. When the coefficient of variation (C.V.) of the model is less than 10%, the experiment is considered to have reliable results. The closer the model’s coefficient of determination R^2^ and the adjusted coefficient of determination R^2^_adj_ are to 1, the better the model’s fit. Precision is the ratio of the effective signal to the noise; a value greater than 4 is considered reasonable [43].

The coefficients of variation for all three response values are less than 10%, indicating that the experiment is reasonably reliable. Furthermore, the coefficients of determination (R^2^) for all three models are very close to 1, suggesting that the surface response models for R_1_, R_2_ and R_3_ provide a good fit. Moreover, the precision of all three models is greater than 4, indicating that the models selected are appropriate.

To examine whether the effects of various factors in the fitted equations were significant, a significance test was conducted on the quadratic polynomial models. Table 12 presents the significance analysis for the quadratic polynomial models of R_1_, R_2_, and R_3_. Significance was assessed using *p*-values and F-values: a smaller *p*-value indicates a higher level of significance (*p* < 0.05 is significant, *p* < 0.01 is highly significant); a larger F-value indicates a greater influence of the factor on the response value.

As shown in Table 12, the impact of various factors on the performance of the asphalt mixtures varied. According to the F-values, the relative significance of the factors affecting Marshall stability follows the order: mixing temperature > wet mixing time > dry mixing time. For −10 °C splitting tensile strength, the order becomes: wet mixing time > dry mixing time > mixing temperature. For residual stability, the order is: wet mixing time > mixing temperature > dry mixing time.

The *p*-values indicate that mixing temperature has a significant effect on Marshall stability, and a significant interaction between mixing temperature and wet mixing time was observed. Both wet mixing time and dry mixing time have highly significant effects on the splitting tensile strength at −10 °C. For residual stability, wet mixing time show a significant impact, and an interaction exist between mixing temperature and dry mixing time.

Furthermore, the lack-of-fit test is not significant, indicating that the model adequately fits the experimental data and that the unexplained variation is not statistically significant.

### 3.3. Model Reliability Analysis

**Figure 8:**
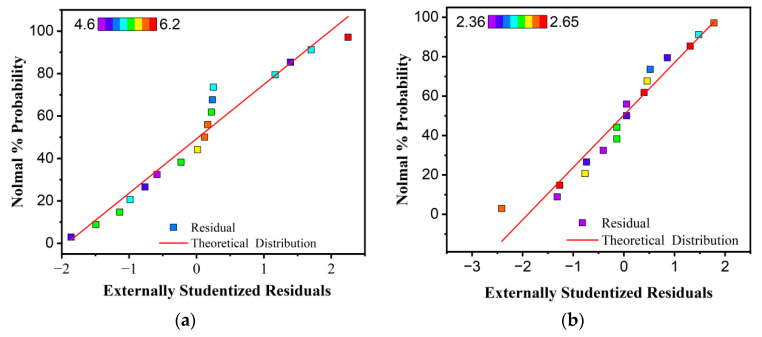

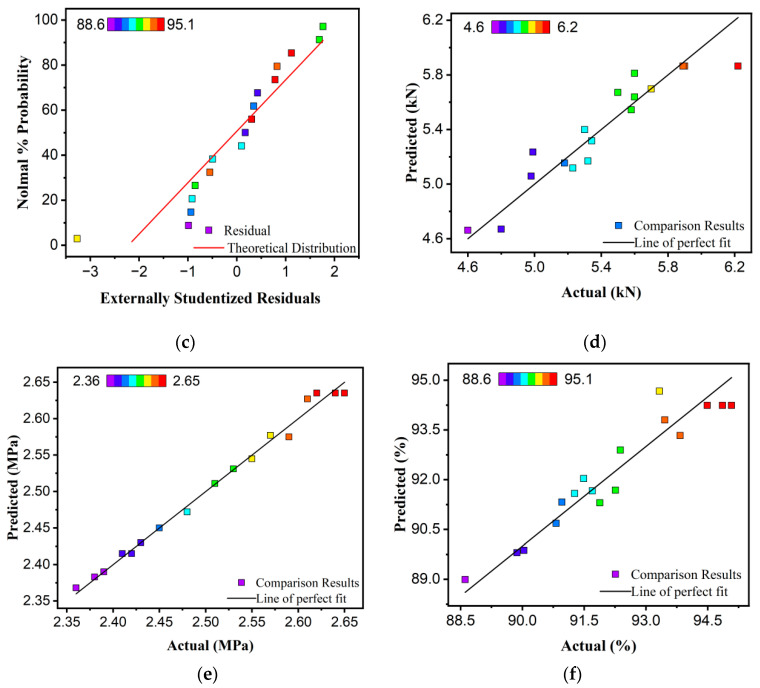
Residual and predicted-versus-actual plots for model validation: (**a**–**c**) residual plots for Marshall stability, splitting tensile strength at −10 °C, and residual stability; (**d**–**f**) predicted versus actual values for the corresponding responses.

The analysis of variance (ANOVA) identified the main effects of each factor and their significant interactions, providing a statistical foundation for the development of predictive models. To further assess the adequacy and robustness of the fitted models, residual plots and predicted-versus-actual plots were generated for the three response variables—Marshall stability, splitting tensile strength at −10 °C, and residual stability—as shown in Figure 8. The residual plots indicate that the residuals are randomly distributed around zero with no discernible pattern, suggesting that the assumptions of homoscedasticity and independence are satisfied. In addition, the normal probability plots show that the residuals closely follow a straight line. The predicted-versus-actual plots further demonstrate that the data points for all three response variables are closely clustered along the diagonal line, indicating strong agreement between experimental and predicted values. This confirms that the developed models possess good fitting accuracy and predictive capability within the investigated parameter range.

### 3.4. Response Surface Interaction Analysis

**Figure 9:**
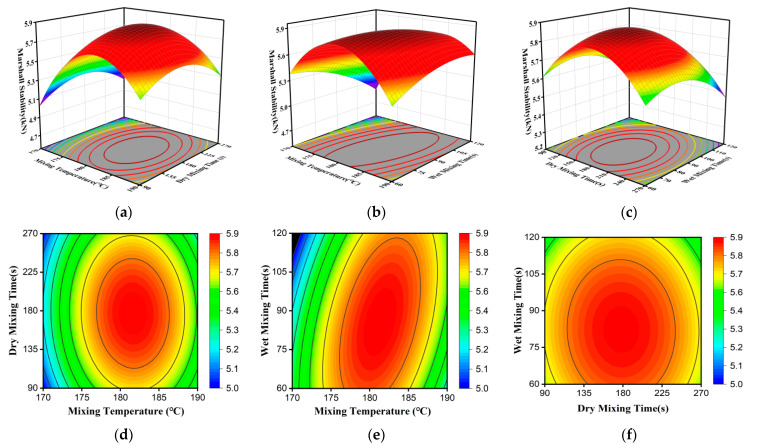
Response surface plots showing the effects of mixing temperature, dry mixing time, and wet mixing time on Marshall stability: (**a**–**c**) 3D response surfaces for temperature–dry time, temperature–wet time, and dry time–wet time; (**d**–**f**) corresponding 2D contour plots (Warmer colors indicate higher response values, whereas cooler colors indicate lower values).

**Figure 10:**
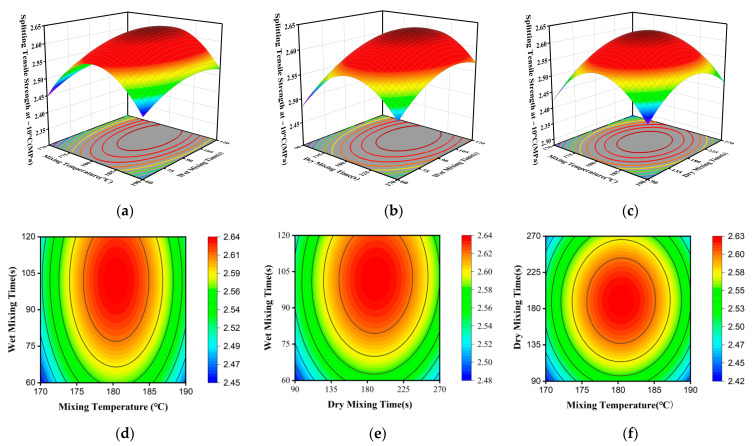
Response surface and contour plots showing the effects of mixing temperature, dry mixing time, and wet mixing time on splitting tensile strength at −10 °C: (**a**–**c**) 3D response surfaces for temperature–wet time, dry time–wet time, and temperature–dry time; (**d**–**f**) corresponding 2D contour plots (Warmer colors indicate higher response values, whereas cooler colors indicate lower values).

**Figure 11:**
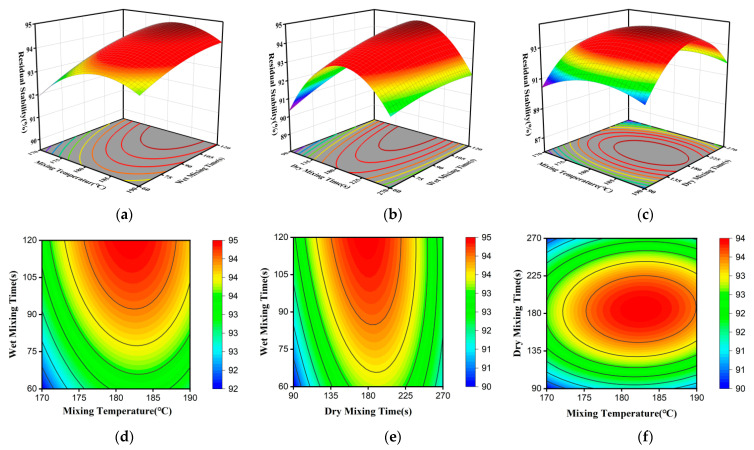
Response surface and contour plots showing the effects of mixing temperature, dry mixing time, and wet mixing time on residual stability: (**a**–**c**) 3D response surfaces for temperature–wet time, dry time–wet time, and temperature–dry time; (**d**–**f**) corresponding 2D contour plots (Warmer colors indicate higher response values, whereas cooler colors indicate lower values).

Significance analysis quantifies the importance of individual factors and their interactions. However, a deeper understanding of these effects requires the construction of three-dimensional (3D) response surfaces and two-dimensional (2D) contour plots. These plots illustrate how factor interactions influence the response variables. The steepness of the response surface reflects the sensitivity of the response to a factor; a steeper surface indicates a more significant influence. Additionally, the shape of the contour lines characterizes the strength of the interaction between two factors, with an elliptical shape indicating a more pronounced interaction [44]. In this study, one variable was held constant while response surfaces and contour plots were generated for the remaining two variables. These plots correspond to Marshall stability, splitting tensile strength at −10 °C, and residual stability, as shown in Figure 9, Figure 10 and Figure 11.

Figure 9 illustrates that the high-temperature performance of the asphalt mixture produced by the dry process is significantly influenced by the mixing temperature. As shown in Figure 9a, when the wet mixing time is 90 s, the Marshall stability exhibits a typical increase–decrease trend with increasing temperature. At a dry mixing time of 180 s, the stability increases from 5.0 kN to a maximum of approximately 5.7 kN as the temperature rises from 170 °C to around 180 °C, followed by a slight decrease to about 5.4 kN at higher temperatures. This trend can be explained by the competing effects of modifier activation and material degradation. As the mixing temperature increases, the modifier undergoes melting and forms a coating on the aggregate surface. A more uniform and continuous modifier film enhances adhesion between the binder and aggregates, leading to a more stable and interlocked aggregate skeleton and improved resistance to deformation at elevated temperatures [45]. However, further temperature increases intensify thermal-oxidative aging of the bitumen and may induce degradation of polymer chains within the modifier. These effects reduce binder ductility and interfacial bonding, ultimately causing a decline in high-temperature performance. A similar trend is observed with increasing dry mixing time at constant temperature. Adequate mixing time is required to achieve effective melting and distribution of the modifier, thereby enhancing its modification efficiency. Nevertheless, excessive dry mixing may intensify thermal exposure and promote degradation processes, offsetting the benefits of improved dispersion.

As shown in Figure 9b, when the dry mixing time is fixed at 180 s, Marshall stability also exhibits an optimum trend with increasing wet mixing time. During wet mixing, the modifier-coated aggregates are progressively covered by a thin asphalt film. Under shear and compressive interactions among aggregates, the modifier diffuses into the binder phase, enhancing its cohesive and adhesive properties. However, once the diffusion process approaches saturation, further extension of wet mixing time yields diminishing returns. Prolonged mixing may instead accelerate binder aging, resulting in a decline in performance. It is also observed that the magnitude of stability improvement depends on the temperature level. At approximately 180 °C, the benefit of increasing wet mixing time is less pronounced than at 190 °C, indicating that higher temperatures promote more efficient modifier diffusion but simultaneously intensify aging effects. Therefore, the high-temperature performance of dry-mix asphalt mixtures is jointly controlled by mixing temperature, dry mixing time, and wet mixing time, with mixing temperature playing a dominant role.

Figure 9d,e show an elliptical shape, demonstrating a significant interaction between mixing temperature and wet mixing time. Furthermore, the contour lines for dry mixing time and wet mixing time are nearly circular, indicating that both factors exert comparable influence on stability, yet their interaction is weak.

As shown in Figure 10, wet mixing time exerts the most significant influence on the −10 °C splitting tensile strength of permeable asphalt mixtures produced via the dry process. When the dry mixing time is 180 s and the mixing temperature remains constant, the split tensile strength at –10 °C continues to increase as the wet mixing time increases. This is because the thickness of the bituminous layer has a significant impact on the low-temperature crack resistance of the bituminous mixture, when it falls below 9 μm, the mixture exhibits poor resistance to cracking [46]. Adequate wet mixing time is crucial for ensuring asphalt film quality. On one hand, it guarantees the formation of asphalt mortar. On the other hand, it accelerates the in situ dispersion and swelling of modifier particles within the matrix asphalt through shear and compression forces between aggregates during mixing. This promotes the formation of a stable spatial network structure, thereby enhancing the modification effect of the binder. When the wet mixing time is held constant, the splitting tensile strength at −10 °C tends to increase initially and then decrease as the mixing temperature rises. The primary reason for this is that, as the mixing temperature increases, the modifier film on the aggregate surface becomes thinner, allowing the modifier to diffuse more readily into the matrix bitumen and thereby enhancing the modification effect. However, excessively high mixing temperatures lead to a reduction in the asphalt’s flexibility, resulting in a decrease in the split tensile strength.

Observing the contour shapes in Figure 10d–f, the contours in Figure 10d exhibit the most pronounced elliptical shape, indicating the most significant interaction between mixing temperature and wet mixing time. The proper matching of these two factors is crucial for enhancing strength. Figure 10e reveals a notable interaction between dry mixing time and wet mixing time. In contrast, the contour lines for mixing temperature and dry mixing time in Figure 10f approach circles, indicating the weakest interaction between these two factors and their relatively independent influence on the results.

Observing the response surfaces in Figure 11a,b, the surface exhibits a pronounced steepening trend along the wet mixing time axis, indicating that the duration of the wet mixing stage is a critical factor determining the water stability of the mixture. Increasing wet mixing time enhances the effective asphalt film thickness on aggregate surfaces and promotes the encapsulation of aggregates by asphalt. This effectively mitigates the action of water at the asphalt-aggregate interface, thereby improving the water stability of asphalt mixtures [47].

When wet mixing time is held constant—either at a fixed dry mixing time or a fixed mixing temperature—both increasing mixing temperature and extending dry mixing time lead to a trend in residual stability that initially increases and then decreases. This behavior can be attributed to the following mechanisms: moderate temperature and mixing conditions promote the formation of a uniform modifier film on the aggregate surface, enhancing interfacial bonding and reducing stripping. However, excessive temperatures accelerate asphalt aging and may induce degradation of modifier molecular chains, thereby weakening water stability. Similarly, moderate dry mixing facilitates the uniform spreading of the modifier film, ensuring it fully encapsulates the aggregates, thereby significantly enhancing the strength of the asphalt film and water stability. Excessively long dry mixing periods can cause the modifier to decompose, diminishing its effectiveness in improving asphalt viscosity and film strength, and resulting in a decline rather than an increase in water stability.

Regarding the interaction between factors, different combinations exhibit varying coupling strengths. The contour lines in Figure 11e exhibit the most pronounced elliptical distortion, indicating a strong synergistic effect between dry mixing time and wet mixing time. A reasonable balance between these two parameters is crucial for enhancing water stability. Figure 11d shows an intermediate interaction between mixing temperature and wet mixing time. In contrast, the contour lines in Figure 11f, corresponding to mixing temperature and dry mixing time, are nearly circular, indicating a weak interaction and relatively independent effects of these two factors.

### 3.5. Best Process and Validation

Since the response surface interaction analysis revealed complex synergistic effects among factors such as mixing temperature, dry mixing time, and wet mixing time, further optimization was conducted to achieve optimal pavement performance. Design-Expert software was employed to systematically optimize these three factors. Considering experimental feasibility, the parameters were rounded to obtain the optimal dry process parameters: a mixing temperature of 182 °C, a dry mixing time of 180 s, and a wet mixing time of 102 s.

**Figure 12:**
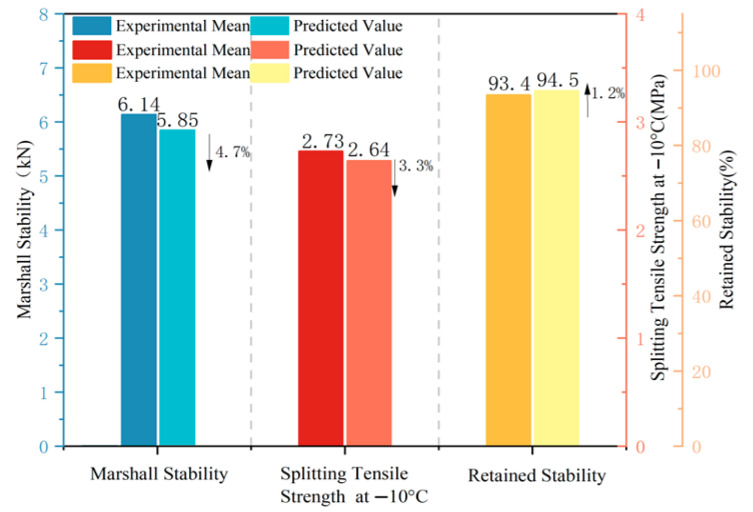
Analysis chart of experimental and predicted values for permeable asphalt mixture properties (An upward arrow indicates that the predicted value is greater than the experimental mean, whilst a downward arrow indicates that the predicted value is less than the experimental mean).

To validate the predictive capability of the developed models, Marshall specimens were prepared using the optimized parameters. Marshall stability, splitting tensile strength, and immersion Marshall stability tests were conducted, with three replicates for each condition. The results are presented in Figure 12. The comparison between predicted and measured values shows that the relative errors for all response variables are within 5%, indicating good agreement. This suggests that the established response surface models provide a reasonable prediction of mixture performance within the studied parameter range.

## 4. Micro-Mechanism Analysis

To investigate the interaction behavior and modification mechanism of high-viscosity modifiers in the dry process, the mixture’s physicochemical properties were characterized using SEM, FM, and Fourier Transform Infrared Spectroscopy (FTIR), thereby elucidating the formation mechanism of the modified structure.

### 4.1. SEM Morphological Analysis

**Figure 13:**
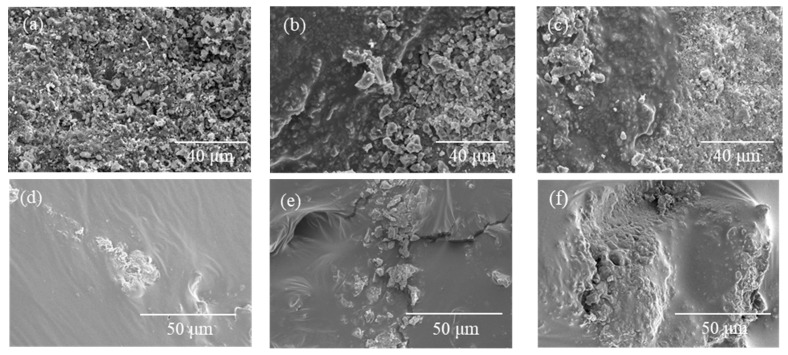
SEM images showing the effect of mixing temperature on HVM dispersion and morphology. Modifier films on aggregate surfaces after dry mixing for 180 s at (**a**) 170 °C, (**b**) 180 °C, and (**c**) 190 °C (3000×). Corresponding mixture morphologies after dry mixing for 180 s and wet mixing for 90 s at (**d**) 170 °C, (**e**) 180 °C, and (**f**) 190 °C (1000×).

To investigate the microstructure of aggregate and mixture surfaces under different mixing conditions, SEM was used to characterize the distribution of the high-viscosity modifier on the aggregate surface and the surface morphology of the asphalt mixture, as shown in Figure 13.

Observation of Figure 13a–c reveals a distinct film structure adhering to the aggregate surface after dry mixing. Under dry mixing conditions at 170 °C, the modifier film structure on the aggregate surface is not prominent; however, as the mixing temperature rises and the melting state of the HVM gradually improves, a modifier film begins to form on the aggregate surface, and the higher the mixing temperature, the thinner the resulting modifier film becomes. From the perspective of the interface between the modifier and the aggregate, the aggregate surface exhibits an uneven structure under an electron microscope. During the mixing process, the molten modifier penetrates the aggregate surface, forming a structure similar to an anchorage. This structure increases the contact area between the modifier and the aggregate, thereby making their bond more stable. This indicates that as the mixing temperature rises within a certain range, the modifier film on the aggregate surface gradually distributes more evenly, and the various performance indicators of the mixture generally show an upward trend. Figure 13d–f show the surface microstructure of the mixture under different mixing conditions. The SEM images reveal wrinkled textures and scattered fine particulate matter. Consistent with previous studies [48,49], these are likely modifier-rich phases or particles formed during wet mixing due to the shearing and interlocking forces between aggregates. As the mixing temperature increases, both the number and distribution of these features become more pronounced, suggesting improved dispersion of the modifier within the asphalt phase. At lower temperatures (e.g., 170 °C), the asphalt phase appears relatively smooth, with limited wrinkling and fewer fine particles, indicating insufficient modification. This observation is consistent with the inferior performance of mixtures prepared at this temperature. In contrast, higher temperatures promote more extensive modifier melting and dispersion, resulting in a more complex microstructure and improved modification effectiveness.

### 4.2. FM Analysis

**Figure 14:**
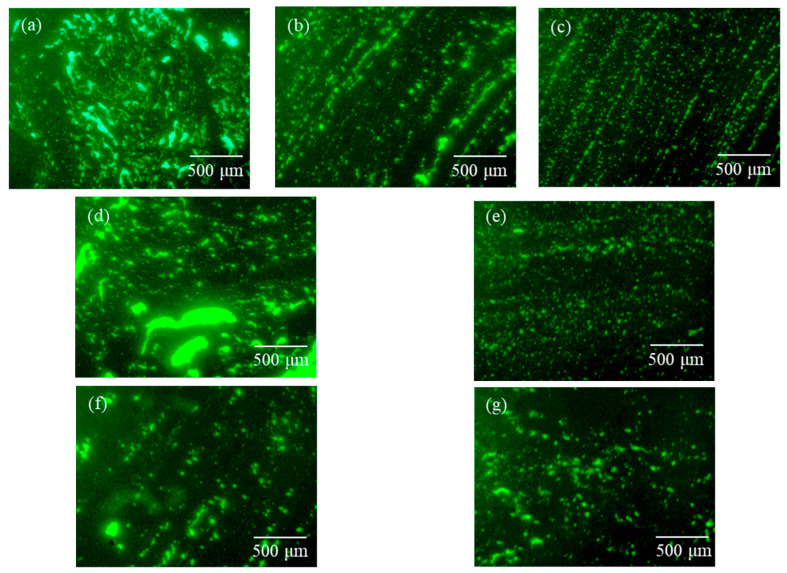
FM images of HVM-modified asphalt extracted under different mixing conditions (400×): (**a**) 170 °C–180s-90s; (**b**) 180 °C–180s-90s; (**c**) 190 °C–180s-90s; (**d**) 180 °C–90s-90s; (**e**) 180 °C–270s-90s; (**f**) 180 °C–180s-60s; (**g**) 180 °C–180s-120s.

While SEM provides effective imaging of the surface after dry mixing, the microstructure of the modifier within the asphalt mixture, particularly in the asphalt binder, requires analysis via FM. Figure 14a–c suggest that increasing the mixing temperature results in finer and more uniformly distributed green fluorescent regions, which may indicate a more homogeneous dispersion of the modifier throughout the base asphalt. Structurally, the morphology of the fluorescent phase appears to transition from elongated, flocculent structures to a more interconnected, network-like appearance. This morphology is reminiscent of the well-developed networks often reported for modifiers prepared via prolonged high-speed shear [50].

Comparing Figure 14d,e seem to indicate that an appropriate dry mixing time facilitates the diffusion of modifiers into the asphalt during wet mixing, forming fine particles and enhancing the modification effect. Furthermore, observation of Figure 14f,g show that, with increasing wet mixing time, the fluorescent phase becomes more clearly defined and the number of dispersed modifier domains increases. This suggests that extending the wet mixing time facilitates the redistribution and dispersion of the modifier within the bitumen. This behavior can be attributed to the swelling of the SBS component within the modifier, which absorbs light fractions from the asphalt and becomes more deformable. Under the combined effects of shear and compression between aggregates, the swollen polymer phase is more readily dispersed within the bitumen matrix. However, excessive wet mixing may also promote phase separation or structural instability, indicating the presence of an optimal mixing duration.

### 4.3. FTIR Analysis

**Figure 15:**
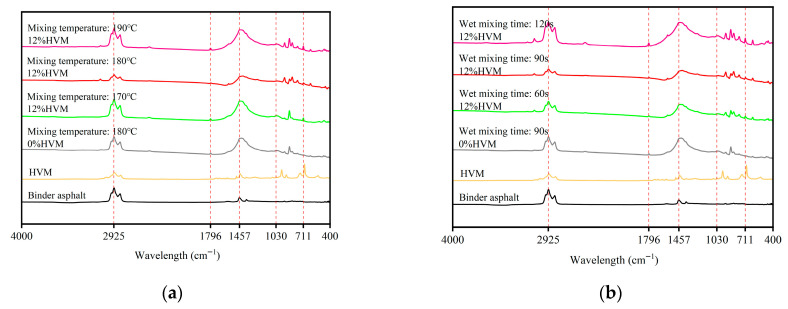
FTIR spectra of dry-process HVM-modified bitumen: (**a**) with a wet mixing time of 90 s; (**b**) at a mixing temperature of 180 °C.

To investigate the modification mechanism of the HVM in the dry-mixing process and the aging behavior of the bituminous binder, the dry mixing time was fixed at 180 s. Bitumen samples were extracted under different mixing temperatures and wet mixing times, and the molecular composition of the bituminous binder was analyzed using a Fourier-transform infrared spectrometer. The FTIR spectra for different mixing temperatures and wet mixing times are shown in Figure 15.

FTIR spectral analysis reveals that all bitumen samples exhibited characteristic alkane absorption peaks at 2925/2852 cm^−1^ (-CH_2_) and 1457/1373 cm^−1^ (-CH_3_). In addition, the HVM exhibits a unique characteristic peak in the 730–675 cm^−1^ range, caused by cis=CH alkenes. This characteristic peak is absent in the base bitumen but appears in the mixed bitumen samples, demonstrating effective physical diffusion between the HVM and the bitumen. As no new absorption peaks are generated in the 1000–600 cm^−1^ range before and after mixing, this indicates that the modification process was primarily physical blending, with no significant chemical reaction. Furthermore, the absorption peaks observed at 1796 cm^−1^ (C=O) and 1030 cm^−1^ (S=O) are associated with the thermal-oxidative aging behavior of the bitumen [51]. Compared with the base bitumen, the extracted binder after mixing exhibits more pronounced peaks, indicating that a certain degree of ageing occurs during the wet mixing process.

The areas of individual absorption peaks were calculated using integration in Origin software, and the indices of characteristic functional groups were determined according to Equation (6) to quantify modifier content and aging degree. The results are presented in Table 13.

I_X_ = A_X_/∑A_i_ × 100 (6)

where I_X_ denotes the index of characteristic functional groups; A_X_ represents the area of characteristic functional groups; ∑A_i_ represents the sum of the areas of functional groups in the range 2000–650 cm^−1^.

A quantitative analysis of FTIR functional group indices revealed the competitive mechanisms involved in the mixing process. The reference sample (0% HVM, 180 °C, 90 s) exhibited higher carbonyl (I_C=O_) and sulphonyl (I_S=O_) indices due to thermal oxidation. Upon the introduction of 12% HVM, both I_C=O_ and I_S=O_ decreased significantly under conditions such as 170–90 and 180–60, demonstrating that HVM can effectively delay the aging of bitumen during the mixing process. However, as the mixing temperature increased or the wet mixing time was extended, these two ageing indices rose again, indicating that more intense mixing conditions exacerbate bitumen ageing. Concurrently, the olefin index (I_=CH_), which reflects the degree of modifier diffusion, rose steadily with increasing temperature and time. This trend indicates that higher temperatures and longer wet mixing durations promote the diffusion of the modifier into the bitumen, thereby contributing to improved mixture performance.

### 4.4. Analysis of the Mechanism of Action of Modifiers

**Figure 16:**
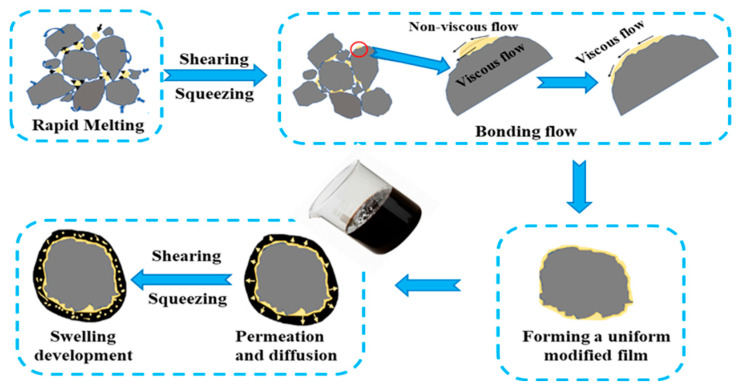
Mixing mechanism between HVM and asphalt in the dry-mixing process.

Based on comprehensive evidence derived from macroscopic performance trends, microstructural observations (SEM and FM) and molecular-level analysis (FTIR), a hypothesis has been supported regarding the four-stage evolution mechanism of HVM during the dry mixing process: rapid melting, viscous flow, permeation and diffusion, and expansion, as shown in Figure 16 [52].

In the initial stage, the modifier particles remain in a relatively solid or semi-molten state due to insufficient thermal energy and limited mixing time. SEM images show clear particle boundaries, while FM observations indicate discrete modifier domains. At this stage, the interaction between the modifier and bitumen is limited, and the olefin index (I_=CH_) obtained from FTIR remains relatively low, suggesting minimal diffusion of the modifier into the asphalt matrix.

In the second stage, with increasing temperature and mixing time, the modifier begins to soften and melt, transitioning into a viscous flow state. This is reflected by the gradual disappearance of particle boundaries in SEM images and a more continuous phase distribution in FM observations. Concurrently, the FTIR results show an increase in the olefin index (I_=CH_), indicating enhanced diffusion of modifier components into the bitumen. At this stage, the modifier starts to actively participate in the structural formation of the mixture.

In the third stage, a permeation–diffusion process dominates, during which the modifier interacts more intimately with the asphalt binder. The FM images exhibit a more uniform and interconnected fluorescence distribution, suggesting improved phase compatibility. FTIR analysis further supports this interpretation, as the continued increase in I_=CH_ indicates ongoing diffusion of modifier molecules. Meanwhile, the reduction in carbonyl (I_C=O_) and sulfoxide (I_S=O_) indices under moderate conditions suggests that the presence of HVM can inhibit the thermal-oxidative aging of bitumen to some extent. This stage corresponds to the optimal parameter range, where performance improvements are most pronounced.

In the final stage, after sufficient diffusion into the bitumen, polymer chains within the modifier extend and absorb light fractions from the base asphalt, leading to a certain degree of swelling. However, due to the limited spatial constraints and the “mix-and-use” nature of the dry process, the extent of swelling is relatively limited.

It should be emphasized that the proposed four-stage mechanism is derived from indirect experimental evidence across multiple scales. While SEM and FM provide morphological and phase-distribution information, and FTIR offers insights into molecular functional groups and aging behavior, these techniques do not directly capture molecular interactions or structural evolution in real time. Therefore, the mechanism should be regarded as an evidence-supported interpretation rather than a definitively established model.

## 5. Conclusions

This study investigated the dry mixing process of permeable asphalt mixtures incorporating a laboratory-prepared HVM through an integrated approach combining RSM, microstructural characterization, and FTIR analysis. As a laboratory-scale study conducted on a single material system, the optimized parameters are system-specific and require further validation before broader application. The primary contribution of this work lies in the proposed methodological framework, which can be extended to the optimization of other dry-mixing systems. The main findings can be categorized as follows:

(1) A multi-response RSM model was successfully established to describe the relationships between mixing parameters and mixture performance. The optimal parameter combination within the investigated range was identified as a mixing temperature of 182 °C, a dry mixing time of 180 s, and a wet mixing time of 102 s. The model showed good agreement with experimental results for most performance indicators.
(2) Analysis of variance indicates that mixing temperature has the most significant influence on high-temperature performance, while wet mixing time plays a dominant role in low-temperature performance and water stability. The interaction between mixing temperature and wet mixing time primarily governs high- and low-temperature performance, whereas the interaction between dry mixing time and wet mixing time predominantly affects water stability.
(3) SEM and FM observations suggest that the modifier undergoes significant morphological evolution during the mixing process. Combined with FTIR results and performance trends, the findings support a four-stage evolution process consisting of softening, melting and flow, permeation–interaction, and structural development. Microstructural analysis confirms that elevated mixing temperatures promote modifier melting, while extended dry and wet mixing times enhance the mechanical shear kneading effect between aggregates. This facilitates a more uniform dispersion of the modifier and the formation of a dense structure within the asphalt matrix.
(4) To further strengthen the mechanistic interpretation and improve performance prediction, future work should incorporate multi-scale characterization. At the binder level, rheological analysis using dynamic shear rheometry (DSR) is recommended to quantify viscoelastic behavior and validate the proposed mechanisms. At the mixture level, advanced performance tests such as wheel-tracking and dynamic modulus should be employed to better capture in-service behavior. This integrated approach would enhance the reliability of translating laboratory optimization results into field applications.

## Figures and Tables

**Table 1:** Technical specifications for high-viscosity modifiers [36].

Technical Indicators	Appearance	Single-Particle Mass (g)	Density (g/cm^3^)	Ash Content (%)
Actual test results	Granular, uniform, plump	0.02	0.81	0.6
Technical requirements	Granular, uniform, plump	≤0.03	≤1	≤1

**Table 2:** Melt flow rate of high-viscosity modifiers.

T/°C	m_nom_ /kg	m/g	t/s	MFR/(g/10 min)	Technical Requirement
190	2.16	0.321	60	3.2	≥2.0

**Table 3:** Absorption peaks and their corresponding functional groups and vibrational modes.

Type	Absorption Peaks/(cm^−1^)	Functional Groups and Vibrational Modes
HVM	2919	-CH_2_- asymmetric stretching vibration
	1448	C=C aromatic ring skeleton vibration
	962	trans =CH olefin out-of-plane bending vibration
	693	cis =CH olefin out-of-plane bending vibration

**Table 4:** Main technical index of coarse aggregate.

Indexes	Test Result	Test Method
Apparent specific density		
9.5–13.2 mm	2.720	
4.75–9.5 mm	2.721	T 0304
2.36–4.75 mm	2.723	
Water absorption (%)		
9.5–13.2 mm	0.54	
4.75–9.5 mm	0.53	T 0307
2.36–4.75 mm	0.79	
Soft stone content (%)	0.6	T 0320
Crushing value of stone (%)	14.8	T 0316
Los Angeles abrasion loss (%)	17.9	T 0317

**Table 5:** Main technical index of fine aggregate.

Indexes	Test Result	Test Method
Apparent relative density	2.726	T 0328
Durability (particles larger than 0.3 mm) (%)	2.6	T 0340
Mud content (percentage of particles less than 0.075 mm)	0.2	T 0333
Sand equivalent (%)	73.4	T 0334
Methylene blue value (g/kg)	1.4	T 0349
Angularity (s)	65	T 0345

**Table 6:** Main technical index of filler.

Indexes	Test Result	Test Method
Apparent relative density	2.415	T 0352
Moisture content (%)	0.4	T 0359
Appearance (%)	73.4	T 0334
<0.60 mm	97.4	T 0351
<0.15 mm	91.8	
<0.075 mm	81.2	
Hydrophilic coefficient	0.91	T 0353
Plasticity index (%)	2.6	T 0354

**Table 7:** Technical properties of 70# A grade asphalt.

Indexes	Test Result	Technical Requirement
Penetration (25 °C, 100 g, 5 s)		
(0.1 mm)	68.5	60–80
Penetration index	−0.75	−1.5–+1.0
Ductility (5 cm/min, 15 °C)		
(cm)	>100	≥100
Softening point (R&B) (°C)	49.0	≥46
Density (g/cm^3^)	1.029	Actual measurement
Paraffin content (%)	1.9	≤2.2
Solubility (%)	99.7	≥99.5
Flash point (°C)	285	≥260
Kinetic viscosity at 60 °C (Pa·s)	193	≥180
After RTFOT		
Mass variation (%)	0.12	−0.8–+ 0.8
Penetration ratio (%)	75.2	≥61
Ductility (5 cm/min, 10 °C) (cm)	6.6	≥6

**Table 8:** Table of Independent Variables and Level Designs.

Level	Factors		
	A	B	C
−1	170	90	60
0	180	180	90
1	190	270	120

**Table 9:** Design proposal and test results for the face-centered cubic structure.

Test Number	A/°C	B/s	C/s	R_1_/kN	R_2_/MPa	R_3_/%
1	180	180	90	5.89	2.64	95.08
2	180	270	90	5.60	2.57	91.49
3	190	270	60	4.98	2.41	90.96
4	180	180	60	5.60	2.59	93.83
5	180	180	90	5.90	2.62	94.49
6	170	180	90	4.99	2.51	92.38
7	170	270	60	5.32	2.39	90.04
8	180	180	90	6.22	2.65	94.86
9	180	180	120	5.70	2.61	93.33
10	170	90	120	4.80	2.42	91.88
11	170	270	120	4.60	2.45	90.82
12	190	270	120	5.34	2.48	92.26
13	170	90	60	5.18	2.36	88.61
14	190	180	90	5.58	2.53	93.46
15	180	90	90	5.50	2.55	91.27
16	190	90	120	5.30	2.43	91.70
17	190	90	60	5.23	2.38	89.87

**Table 10:** Analysis of variance for regression models.

Response	Sequential *p*-Value	Lack of Fit *p*-Value	Adjusted R-Squared (R^2^adj)	Predicted R-Squared (R^2^)
R_1_	0.0029	0.4428	0.7258	0.1075
R_2_	<0.0001	0.6817	0.981	0.9482
R_3_	<0.0001	0.6817	0.981	0.9482

**Table 11:** Model validity testing.

Response	C.V.%	R^2^	R^2^_adj_	Precision
R_1_	4.07	0.8800	0.7258	7.1413
R_2_	2.51	0.9917	0.9810	25.8576
R_3_	0.91	0.9095	0.7931	8.8508

**Table 12:** Significance analysis of the R_1_, R_2_, and R_3_ using the secondary polynomial models.

Model	R_1_		R_2_		R_3_	
Parameter	F-Value	*p*-Value	F-Value	*p*-Value	F-Value	*p*-Value
A	4.94	0.0458	5.51	0.0513	2.92	0.1311
B	0.0576	0.8173	14.11	0.0071	0.7207	0.424
C	0.6654	0.4415	37.26	0.0005	6.37	0.0396
AB	0.0557	0.8202	0.2756	0.6158	0.299	0.0315
AC	6.09	0.043	0	1	0.1479	0.7119
BC	0.0056	0.9422	0.2756	0.6158	1.62	0.2434
A^2^	12.55	0.0094	189.82	<0.0001	3.1	0.1219
B^2^	2.46	0.1608	79.51	<0.0001	22.77	0.002
C^2^	0.6775	0.4376	16.45	0.0048	0.2233	0.6509
Lack-of-Fit	1.52	0.4428	0.6887	0.6817	10.86	0.0865

**Table 13:** Characteristic functional group index.

Functional Group Index	180–90 (%)	170–90 (%)	180–60 (%)	180–60 (%)	180–120 (%)	190–90 (%)
I_C=0_	0.9	0.82	0.68	0.64	1.26	1.00
I_S=0_	2.88	0.89	0.96	1.25	1.97	1.66
I_=CH_	/	0.73	0.85	1.07	1.08	0.89

## Data Availability

The original contributions presented in this study are included in the article. Further inquiries can be directed to the corresponding author.
